# mGlu5 Receptor Blockade Within the Nucleus Accumbens Shell Reduces Behavioral Indices of Alcohol Withdrawal-Induced Anxiety in Mice

**DOI:** 10.3389/fphar.2018.01306

**Published:** 2018-11-13

**Authors:** Kaziya M. Lee, Michal A. Coelho, MacKayla A. Class, Kimberly R. Sern, Mark D. Bocz, Karen K. Szumlinski

**Affiliations:** ^1^Department of Psychological & Brain Sciences, University of California, Santa Barbara, Santa Barbara, CA, United States; ^2^Department of Molecular, Cellular, and Developmental Biology and the Neuroscience Research Institute, University of California, Santa Barbara, Santa Barbara, CA, United States

**Keywords:** binge drinking, adolescence, group 1 metabotropic glutamate receptors, anxiety, depression, alcoholism, nucleus accumbens

## Abstract

Withdrawal from binge-drinking increases negative affect, coinciding with increased expression of the metabotropic glutamate receptor 5 (mGlu5) within the shell of the nucleus accumbens (AcbSh). Supporting a causal-effect relationship, systemic treatment with the mGlu5 receptor antagonist MTEP [3-((2-Methyl-4-thiazolyl)ethynyl)pyridine] is anxiolytic in binge-drinking adult and adolescent mice. Here, we employed neuropharmacological approaches to examine the functional relevance of AcbSh mGlu5 for behavioral indices of alcohol withdrawal-induced hyper-anxiety. Adult (PND 56) and adolescent (PND 28) male C57BL/6J mice consumed alcohol under modified Drinking-in-the-Dark procedures (10, 20, and 40% alcohol v/v) for 14 days. At an alcohol withdrawal time-point when mice manifest robust behavioral signs of hyper-anxiety (1 and 28 days withdrawal for adults and adolescents, respectively), mice were infused intra-AcbSh with 0, 1 or 10 μg MTEP and then affect was assayed in the light-dark shuttle box, marble-burying and forced swim tests. Brain tissue was collected to evaluate changes in Egr1 (early growth response protein 1) induction to index AcbSh neuronal activity. As expected, alcohol-experienced mice exhibited behavioral signs of hyper-emotionality. The anxiolytic effects of intra-AchSh MTEP were modest, but dose-dependent, and varied with age of drinking-onset. In adult-onset mice, only the 1 μg MTEP dose reduced withdrawal-induced hyper-anxiety, whereas only the higher dose was effective in adolescent-onset animals. MTEP reduced Egr1 expression within the AcbSh, irrespective of alcohol drinking history or age of drinking-onset. However, only the high MTEP dose reduced Egr1 expression in adolescent-onset binging mice. These results implicate AcbSh mGlu5 in modulating alcohol withdrawal-induced negative affect and suggest age differences in the neurobiological effects of alcohol withdrawal and behavioral responsiveness to mGlu5 blockade within the AcbSh.

## Introduction

Binge-drinking is defined as a pattern of consumption that elevates blood alcohol concentrations (BAC) to ≥80 mg/dl, which equates to approximately 4–5 drinks in a 2-h period (National Institute on Alcohol Abuse Alcoholism [NIAAA], 2004). In both humans and laboratory animal models, a history of binge alcohol-drinking augments symptoms of negative affect and dysphoria during periods of abstinence (e.g., Hasin and Grant, 2002; Grant et al., 2004; Squeglia et al., 2009; Lee et al., 2015, 2016, 2017a,b, 2018) and this withdrawal-induced negative affect is theorized to drive the negative reinforcing properties of alcohol (Becker, 2012; Tunstall et al., 2017). While both adults and adolescents engage in binge-drinking, this pattern of alcohol consumption is especially prevalent amongst adolescents and young adults (National Institute on Alcohol Abuse Alcoholism [NIAAA], 2017). Adolescents typically consume larger quantities of alcohol than adults, yet adolescents appear to be less sensitive to the negative consequences of acute intoxication (e.g., locomotor incoordination and sedation) and experience fewer ‘hangover’-like symptoms, including withdrawal-induced negative affect (White et al., 2002; Doremus et al., 2003; Varlinskaya and Spear, 2004; Spear and Varlinskaya, 2005).

Indeed, age-related differences in the manifestation of alcohol withdrawal-induced negative affect are apparent in murine models of binge-drinking (Lee et al., 2016, 2017c). More specifically, mice with a history of adult-onset binge-drinking exhibit increased behavioral signs of hyper-anxiety during early withdrawal (i.e., 24 h post-drinking), the magnitude and duration of which varies as a function of the amount of alcohol consumed (Lee et al., 2015, 2016, 2017a,c, 2018). In contrast, mice with a history of adolescent-onset binge-drinking do not exhibit these signs of hyper-anxiety during early alcohol withdrawal (Lee et al., 2016, 2017a); rather, negative affect incubates with the passage of time during alcohol abstinence, manifesting robustly later in adulthood (Lee et al., 2017b,c). These latter findings are in-line with the epidemiological literature demonstrating latent and enduring emotional disturbances in individuals with a history of problem drinking during adolescence (e.g., Hasin and Grant, 2002; Grant et al., 2004; Hasin et al., 2007; Squeglia et al., 2009) and argue that binge-drinking during adolescence perturbs the development of neural circuits controlling emotionality.

In mouse models, withdrawal from a chronic history of alcohol consumption up-regulates the expression of the metabotropic glutamate receptor 5 (mGlu5) within the nucleus accumbens shell (AcbSh), present at as early as 24-h withdrawal (Cozzoli et al., 2012; Lee et al., 2016). This increase in expression coincides with the manifestation of behavioral indices of negative affect (Lee et al., 2016, 2017a). Consistent with a purported role for mGlu5 in regulating anxiety (reviewed in Bergink et al., 2004; Swanson et al., 2005; Simon and Gorman, 2006), systemic treatment with negative allosteric modulators of mGlu5 alleviates signs of withdrawal-induced anxiety in rodents (Kotlinska and Bochenski, 2008; Kumar et al., 2013; Lee et al., 2017a,b). The AcbSh is a component of the extended amygdala macrocircuit (Alheid, 2003), the dysregulation of which is theorized to be a critical driver of the negative reinforcing properties of alcohol (e.g., Gilpin and Koob, 2008; Tunstall et al., 2017). Given that: (1) mGlu5 expression is up-regulated during withdrawal from binge-drinking (Cozzoli et al., 2012; Lee et al., 2016); (2) inhibiting AcbSh activity produces an anxiolytic effect (Lopes et al., 2012) and (3) mGlu5 inhibition within the AcbSh reduces signs of opioid withdrawal-induced anxiety (Radke and Gewirtz, 2012; Lou et al., 2014), the present study tested for a cause-effect relation between the coincident increase in AcbSh mGlu5 expression and hyper-emotionality during binge-alcohol withdrawal (Lee et al., 2016, 2017c). For this, mice with a history of binge-drinking during either adolescence or adulthood were infused intra-AcbSh with the mGlu5 negative allosteric modulator 3-[(2-Methyl-1,3-thiazol-4-yl)ethynyl]pyridine (MTEP) at a time post-alcohol consumption when adult- and adolescent-onset drinking mice exhibit robust behavioral signs of negative affect (i.e., during early and protracted withdrawal, respectively; Lee et al., 2016, 2017b, 2018). Here, we report that intra-AcbSh MTEP produces moderate anxiolytic effects, irrespective of the age of binge-drinking onset. However, consistent with our prior results for systemic MTEP treatment (Lee et al., 2017a), age-related differences exist with respect to the sensitivity to MTEP’s anxiolytic effects.

## Materials and Methods

The binge-drinking and behavioral testing procedures used in this study were identical to those employed previously in our laboratory (Lee et al., 2016, 2017b) and are briefly summarized below. All procedures were conducted in compliance with the National Research Council (U.S.) (2011) (United States). Committee for the Update of the Guide for the Care and Use of Laboratory Animals., Institute for Laboratory Animal NIH Publication No. 80–23 and approved by the Institutional Animal Care and Use Committee (IACUC) of the University of California, Santa Barbara.

### Subjects

The subjects in this study were male C57BL/6J mice, purchased from The Jackson Laboratory (Sacramento, CA, United States). Mice were allowed 7 days to acclimate to our vivarium and were either PND 28 (adolescents) or PND 56 (adults) at the onset of our binge-drinking procedures. Animals were identified using small animal ear tags (Stoelting, Wood Dale, IL, United States) and housed in age-matched groups of 4 in a climate-controlled vivarium under a reverse light/dark cycle (lights off at 10:00 h). Food and water were available *ad libitum*, except during the 2-h alcohol-drinking period. The study design consisted of 2 age of drinking-onset groups (adults versus adolescents), 2 drinking groups (alcohol versus water), and 3 intra-AcbSh treatment groups (0, 1 or 10 μg MTEP). The sample size was 12 per group.

### Modified Drinking-in-the-Dark (DID) Procedures

Half of the animals from each age of drinking-onset group were subjected to 14 consecutive days of binge-drinking under 3-bottle DID procedures. Control animals received water only. As in our more recent studies (Lee et al., 2016, 2017a,b,c, 2018) alcohol-access was restricted to 14 days for all animals, which corresponds to the approximate duration of early-mid adolescence in mice (Spear, 2000). Each day, animals were separated into individual drinking cages, allowed 45 min to habituate to the drinking cage and then given concurrent access to 10, 20, and 40% (v/v) unsweetened alcohol solutions for 2 h, beginning 3 h into the circadian dark cycle (Rhodes et al., 2005). The amount of alcohol consumed each day was calculated by bottle weight immediately before and after the drinking period and expressed as a function of the animal’s body weight (g/kg). All animals, both alcohol and water drinkers, were weighed three times per week throughout the drinking period.

Submandibular blood samples were collected from all alcohol-drinking animals on day 11 of alcohol presentation, immediately upon conclusion of the 2-h drinking period. The scheduling of the blood sampling was selected to ensure that the animals’ intakes had stabilized, while also allowing ample time for recovery prior to behavioral testing. BAC was determined using an Analox alcohol analyzer (model AM1, Analox Instruments USA, Lunenburg, MA, United States).

### Behavioral Testing

Based on our previous work showing age-related differences in the emergence of a withdrawal phenotype (Lee et al., 2016, 2017c), all animals were behaviorally tested at PND 70 (i.e., all animals were adults at the time of behavioral testing). The behavioral testing battery consisted of the light-dark box, followed by the marble burying test, and concluded with the Porsolt forced swim test (FST). Counterbalancing of tests was not employed based on our IACUC’s recommendation that no subsequent testing immediately follow the FST within the same day, to allow animals sufficient time to dry off and recover. The specific procedures are detailed in our published work (see Lee et al., 2016, 2017a,b,c, 2018). While elevated marble-burying is argued to reflect compulsive digging rather than anxiety-like behavior (e.g., Thomas et al., 2009), marble-burying can be attenuated by non-hypnotic doses of anxiolytics (c.f., Albelda and Joel, 2012). Importantly for this study, increased marble-burying behavior during alcohol withdrawal correlates with the expression of other behavioral indices of anxiety or negative effect assayed using light-dark shuttle-box and forced swim and all three of these assays have been consistently sensitive to the effects of our 14-day binge-drinking procedure on alcohol withdrawal-induced negative affect (e.g., Lee et al., 2016, 2017b).

#### The Light/Dark Shuttle Box

This test was employed to assay anxiety-like behaviors (Crawley, 1985; Bourin and Hascoet, 2003) and involved placing mice into a polycarbonate box (46 cm long × 24 cm high × 22 cm wide), which was equally subdivided into a white, uncovered compartment and a black, covered compartment. The compartments were separated by a central divider with an opening and the testing commenced with the animals on the dark side and the latency to enter the light side, number of light-side entries, and total time spent in the light side of the shuttle box were recorded during the 15-min trial using Any-maze tracking software^TM^ (Stoelting Co., Wood Dale, IL, United States). General locomotor activity was also assessed by measuring the total distance traveled during the trial. We also employed the marble-burying test as an alternate index of anxiety (Nicolas et al., 2006).

#### Marble Burying

In this test, 10 square glass pieces (2.5 cm 2 × 1.25 cm tall) were placed in the animals’ home cage, 5 at each end. The total number of marbles with at least 75% of their surface area covered with sawdust bedding were counted at the end of the 20-min trial. Each animal was video recorded and an experimenter blind to the treatment of the mice scored the videos using a stopwatch to determine the latency to begin burying marbles and the total time spent burying.

#### The Porsolt Forced Swim Test

In this assay, each mouse was placed into an 11-cm diameter cylindrical container and the latency to first exhibit immobility (defined as no horizontal or vertical displacement of the animal’s center of gravity for ≥5s), total time spent immobile, and the numbers of immobile episodes were monitored throughout the entire 6-min trial period using Any-maze^TM^ tracking software. Behavioral testing was conducted during the animals’ circadian dark phase.

### Surgical Procedures

Both adult and adolescents underwent surgery at the same age, approximately 3 weeks prior to behavioral testing (PND 42–48). Thus, surgery occurred prior to the 14-day drinking period in adults and afterward in adolescents. In addition to maintaining consistency in the surgery timing across age groups, this also allowed us to avoid the practical limitations of performing craniotomies on juvenile mice. Surgical procedures were similar to those conducted previously in our laboratory (e.g., Cozzoli et al., 2012; Lum et al., 2014). Under 1–1.5% isoflurane anesthesia, animals were implanted with bilateral indwelling guide cannulae (20-gauge, 10 mm long) positioned 2 mm above the AcbSh, based on coordinates from the mouse brain atlas of Paxinos and Franklin (2004): AP: + 1.3; ML: ± 0.5 mm; DV: -2.3 mm from Bregma. Cannulae were secured to the skull surface using dental resin and dummy cannulae (24 gauge; 10 mm long) were placed inside the guide cannulae to prevent externalization. Animals were allowed to recover for a minimum of 7 days before further experimentation.

### Intracranial Drug Infusion Procedures

MTEP (4 and 40 μg/μl; Sigma-Aldrich; St. Louis, MO, United States) was dissolved in sterile water and infused at a volume of 0.25 μl/side, such that animals were infused with either 1 or 10 μg/side. These doses were selected based on prior neuropharmacological studies by our group demonstrating effectiveness at reducing binge-drinking in mice (Cozzoli et al., 2009, 2014a). To be consistent with our prior neuropharmacological studies of binge-drinking (Cozzoli et al., 2009, 2012), sterile water served for control infusions (0 μg MTEP). On the day of behavioral testing (PND 70), microinjectors (33-gauge, 12 mm long) were lowered into the guide cannulae and mice were infused with their assigned MTEP dose at a rate of 0.25 μl/min for 60 s. Injectors were left in place for an additional 60 s to allow for drug diffusion. Animals were then returned to their home cages for approximately 15 min before the start of testing, to allow for maximal drug efficacy.

### Immunohistochemistry

Within 5 min of the completion of behavioral testing, animals were euthanized with an overdose of Euthasol^®^ (0.3 ml total volume; Virbac AH, Fort Worth, TX, United States) and transcardially perfused with phosphate-buffered saline (PBS), followed by 4% paraformaldehyde. Brains were removed and post-fixed for 24 h in 2% paraformaldehyde in PBS, then cold-stored in cryoprotectant (30% ethylene glycol, 30% glycerol in PBS to prevent ice crystal formation) until slicing. Tissue was sectioned (40 μm) along the coronal plane on a vibratome at the level of the striatum (as depicted in Paxinos and Franklin, 2004). Immunohistochemistry was performed to detect expression of Egr1, an immediate early gene (IEG) encoded inducible transcription factor commonly used as a marker of localized brain activation in laboratory animals (Lee, 2014). Egr1 induction is mediated by MAP kinase-activated transcription factors Elk-1, SAP-1, and SAP-2. Elk-1 then associates with CREB binding protein and serum response factor to bind at the serum response element of the promoter region (Clarkson et al., 1999). Egr1 has higher constitutive expression compared to other common IEGs such as c-Fos, making it sensitive to both increases and decreases in expression (Herdegen and Leah, 1998). Thus, changes in Egr1 expression were used to determine the effects of alcohol withdrawal and MTEP treatment on neuronal activation within the AcbSh.

**FIGURE 1:**
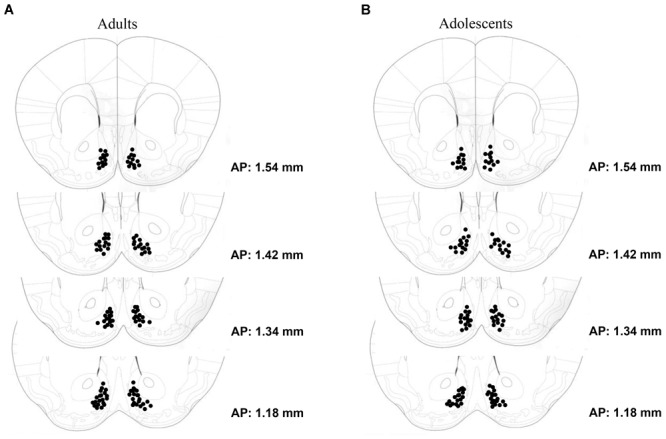
Depiction of the microinjector tip placements within the AcbSh of **(A)** adult-onset (N = 63) and **(B)** adolescent-onset (N = 59) drinking groups.

Tissue sections were stained using standard immunohistochemical procedures, as described in Lee et al. (2015). In brief, sections were treated with 0.25% Triton X-100 (Sigma #X-100, St. Louis, MO, United States) and 5% dimethyl sulfoxide (Sigma D-5879), and then incubated for 1 h in 20% normal horse serum (NHS; Sigma G6767) + 1% bovine serum albumin (BSAFract V; Fisher Scientific, Los Angeles, CA, United States, BP1605-100) to block non-specific binding. Slides were then incubated for 24 h in a rabbit Egr1 primary antibody 1:1000 (c-19 anti-Egr1; Santa Cruz Biotechnology, Santa Cruz, CA, United States) + 0.5% Triton X-100 + 1% NHS. Next, sections were incubated for 1 h in the secondary antirabbit IgG antibody (Vector Laboratories BA110, Burlingame, CA, United States), and for 30 min in the avidin–biotin horseradish peroxidase complex (Elite Vectastain Universal ABC Kit, Vector Laboratories PK6200, Burlingame, CA, United States). Staining was visualized using the chromogen 3,3_-diaminobenzidine (DAB) (Vector Laboratories Peroxidase Substrate Kit SK-4100). Following staining, sections were dehydrated and cover-slipped. Slides were viewed with a Nikon Eclipse E800 microscope equipped with a Hamamatsu CCD camera (model C4742-95). Images were acquired at 40× magnification using MetaMorph^®^ software (Molecular Devices, Sunnyvale, CA, United States). Egr1 expression was analyzed in the AcbSh, as well as the adjacent core subregion (AcbC) for comparison. DAB staining intensity was quantified using ImageJ (NIH Image, National Institutes of Health, Bethesda, MD, United States^1^), using the ‘mean gray’ function. The mean gray intensity value of the entire image was divided by an internal background control value for each animal obtained from a region lacking any Egr1+ cells, yielding a normalized measure (arbitrary units) of staining intensity (similar to as described in Hartig, 2013; Jensen, 2013; Nguyen et al., 2013). A left- and right-side measurement was summed for each animal. In addition to an analysis of staining, slides were also examined for proper microinjector placement. Only animals with injector cannulae located within the boundaries of the AcbSh were included in the statistical analyses of the data (see Figure 1).

### Statistical Analysis

A mixed-design, repeated measures analysis of variance (ANOVA) was used to analyze intake data for all alcohol-drinking animals to assess age differences in alcohol consumption across the 14-day drinking period. A Pearson’s correlational analysis was conducted to determine the relationship between alcohol intake and resulting BACs sampled on day 11 of drinking. Two-way ANOVAs were used to analyze the behavioral data and immunohistochemistry, with Drinking (alcohol or water) and Treatment (MTEP 10 μg/side, 1 μg/side, or vehicle) as factors. Tukey–Kramer multiple comparison tests were used to further deconstruct group differences for ANOVAs yielding significant findings. Tukey–Kramer tests were also applied to data for which the results of the ANOVAs failed to indicate significant interactions between our independent variables to address *a priori* hypotheses regarding specific drug-related differences in behavior. This was done as Tukey–Kramer tests provide conservative protection against Type I error while maximizing statistical power (Kramer, 1956; Hayter, 1984; Cardinal and Aitken, 2006), and these tests are particularly well-suited to studies that contain multiple *a priori* comparisons of interest. Based on the hypotheses in this study, MTEP-treated animals were compared to vehicle-treated animals within each drinking group, as we predicted that MTEP treatment would have an anxiolytic effect, and vehicle-treated water-drinkers were compared with vehicle-treated alcohol-drinkers to confirm the presence of withdrawal-induced negative affect. We predicted that MTEP would reduce Egr1 expression in the AcbSh compared to vehicle treatment in both water- and alcohol-drinkers and the effect of alcohol withdrawal on Egr1 expression among vehicle-treated animals was also assessed. Statistical outliers were identified for each test using the ±1.5×IQR rule and excluded from analysis. All data depicted in figures represent mean ± SEM of the number of the number of animals indicated in parentheses; *p*-values less than 0.05 were considered to be significant for all tests. Tukey–Kramer analyses were performed in Microsoft Excel using add-on StatPlus^6.0^ and all other calculations and analyses were performed using SPSS v.21 statistical software.

## Results

### Animal Exclusion

Animals were omitted entirely from data analysis based on incorrect microinjector placement, difficulties encountered during staining, and attrition due to surgical complications, resulting in final samples sizes of *n* = 9–11 per group. Data identified as statistical outliers were also omitted from analysis, resulting in the exclusion of 0–2 animals per test.

### Alcohol Intake

**FIGURE 2:**
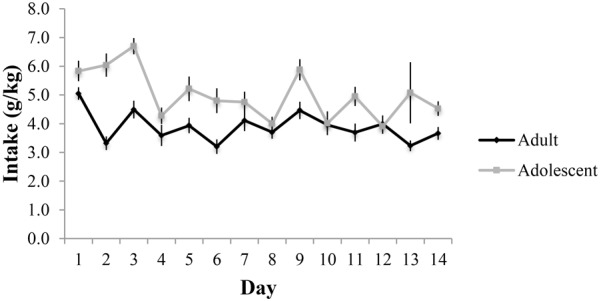
Alcohol consumption across the 14-day drinking period in adult- and adolescent-onset alcohol drinkers.

Adults consumed an average of 3.88 ± 0.20 g/kg and adolescents an average of 5.00 ± 0.21 g/kg across the 14-day drinking period (Figure 2). These data are comparable to that observed in our prior reports of surgery-naïve mice (e.g., Lee et al., 2016, 2017a,b,c), suggesting that the surgical procedures did not significantly alter subsequent alcohol intake in adult mice. The repeated-measures ANOVA showed a significant effect of age of drinking-onset, which reflected greater alcohol intake, overall, in adolescent versus adult mice [*F*(1,58) = 14.93, *p* < 0.001]. On day 11 of drinking, adults consumed an average of 3.69 ± 0.31 g/kg resulting in an average BAC of 69.93 ± 3.17 mg/dl and adolescents consumed an average of 4.94 ± 0.34 g/kg resulting in an average BAC of 78.29 ± 3.01 mg/dl. Although the correlation between alcohol intake and resulting BAC on day 11 did not quite reach significance, there was a strong statistical trend for a positive relationship (*r* = 0.242, *p* = 0.062).

### Intra-AcbSh MTEP Effects on the Behavior of Adult-Onset Drinking Mice During Early Alcohol Withdrawal

#### Light-Dark Box

**FIGURE 3:**
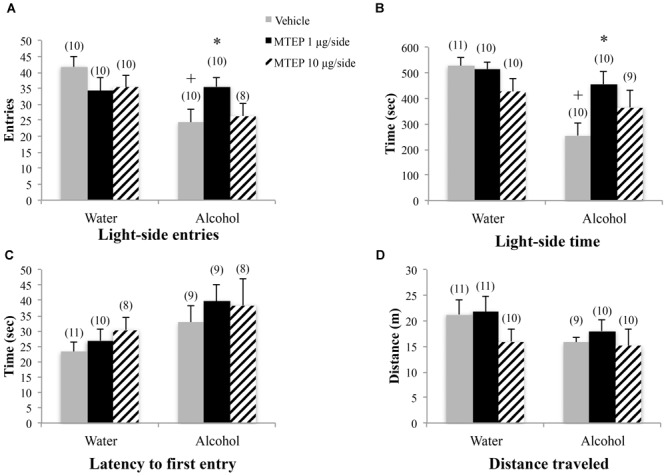
Effects of intra-AcbSh MTEP and adult-onset alcohol consumption upon behavior in the light-dark box. **(A)** Vehicle-infused adult-onset alcohol-drinking mice made fewer light-side entries than their water controls. Infusion with 1 μg/side MTEP increased the number of light-side entries exhibited by alcohol-drinking mice, compared to vehicle-infused controls. **(B)** Similarly, vehicle-infused alcohol-drinking mice also spent less time on the light side, compared to water controls, and 1 μg/side MTEP significantly increased light-side time in alcohol-drinking mice, compared to vehicle infusion. **(C)** There were no significant group differences in the latency to first light-side entry or **(D)** the distance traveled during the 15-min test. ^∗^p < 0.05 treatment effect within same drinking group, ^+^p < 0.05 drinking effect within same intracranial treatment group.

The ANOVA revealed a significant treatment × drinking interaction regarding the number of light-side entries [*F*(2,54) = 3.31, *p* = 0.044; Figure 3A]. Tukey–Kramer *post hoc* comparisons showed that in vehicle-infused animals, adult-onset alcohol-drinking mice made fewer light-side entries, compared to their water controls (*p* = 0.001). Infusion of 1 μg/side MTEP significantly increased the number of light-side entries in the alcohol-drinking mice, compared to vehicle infusion (*p* = 0.039). In contrast, this low MTEP dose did not alter the number of light-side entries in water drinkers and the 10 μg/side dose did not alter this measure in either alcohol- or water-drinking mice (*p’*s > 0.10). There was a similar treatment × drinking interaction in the time spent on the light side [*F*(2,54) = 3.50, *p* = 0.037; Figure 3B] and *post hoc* comparisons showed that in vehicle-treated animals, alcohol-drinking mice spent less time on the light side, compared to their water-drinking counterparts (*p* < 0.001). In alcohol-drinking mice specifically, 1 μg/side MTEP significantly increased the time spent on the light side, compared to vehicle infusion (*p* = 0.004). However, there was no effect of 1 μg/side MTEP in water-drinking mice and no effect of the higher dose in either drinking group (*p*’s > 0.10). There was a significant main effect showing that, overall, alcohol-drinking animals had a longer latency to first entry compared to water-drinking animals [*F*(1,49) = 5.16, *p* = 0.028; Figure 3C]. However, there was no main effect or interaction related to intracranial treatment (*p*’s > 0.10). There were also no main effects or interaction observed for total distance traveled during testing (*p*’s > 0.10; Figure 3D).

#### Marble Burying

**FIGURE 4:**
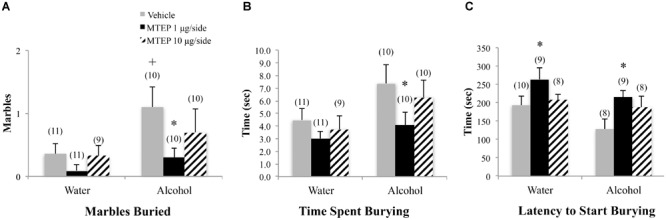
Effects of intra-AcbSh MTEP and adult-onset alcohol consumption upon behavior in the marble-burying test. **(A)** Vehicle-infused adult-onset alcohol-drinking mice buried more marbles than their water controls and infusion of 1 μg/side MTEP significantly reduced the marble burying exhibited by alcohol-drinking mice, compared to their vehicle-infused controls. **(B)** Vehicle-infused alcohol-drinking mice trended toward more time spent burying than water controls (p = 0.061) and 1 μg/side MTEP significantly reduced the time spent burying by alcohol-drinking mice, compared to vehicle treatment. **(C)** Vehicle-infused alcohol-drinking mice also trended toward a shorter latency to start burying than water-drinking controls and 1 μg/side MTEP significantly increased this latency, relative to vehicle infusion, irrespective of drinking history. ^∗^p < 0.05 treatment effect within same drinking group, ^+^p < 0.05 drinking effect within same intracranial treatment group.

A significant main effect showed that adult-onset alcohol-drinking animals buried more marbles overall compared to water control animals [*F*(1,55) = 5.59, *p* = 0.022; Figure 4A]. Although the interaction term was not significant, an inspection of Figure 4A suggested a selective effect of the 1 μg/side MTEP dose upon the marble-burying of adult-onset alcohol-drinking mice. Tukey–Kramer comparisons were then conducted and the results showed that among vehicle-infused animals, adult-onset alcohol-drinking mice buried more marbles, compared to their water controls (*p* = 0.023). Alcohol-drinking mice treated with 1 μg/side MTEP buried fewer marbles, compared to their respective vehicle-infused group (*p* = 0.016), but there was no effect of 1 μg/side MTEP in water-drinking animals and no effect of 10 μg/side in either the water or alcohol group (*p*’s > 0.10). A similar main effect showed that alcohol-drinking animals also spent more time marble burying overall, compared to water-drinking animals [*F*(1,55) = 5.90, *p* = 0.018; Figure 4B]. As inspection of Figure 4B suggested that MTEP exerted a dose-dependent effect on the time spent marble-burying, we re-analyzed the data and detected a trend toward more time spent burying in alcohol- versus water-drinking mice infused with vehicle (*p* = 0.061). The 1 μg/side MTEP dose reduced the time spent marble burying by alcohol-drinking mice, compared to vehicle-infused controls (*p* = 0.037). However, the low MTEP dose did not affect this measure in water-drinking animals and the 10 μg/side MTEP dose did not influence the time burying, irrespective of drinking history (*p*’s > 0.10). A significant main effect showed that alcohol-drinking animals had a shorter latency to begin burying overall compared to water-drinking animals [*F*(1,46) = 4.55, *p* = 0.038; Figure 4C] and also revealed a significant effect of intracranial treatment [*F*(1,46) = 4.97, *p* = 0.011]. Tukey–Kramer comparison analyses showed that alcohol drinking did not alter the latency to begin burying among vehicle-infused animals, relative to water-drinking controls (*p* > 0.10). However, in both water- and alcohol-drinking animals, 1 μg/side MTEP significantly increased latency to begin burying (*p* = 0.046 and *p* = 0.021, respectively), while the 10 μg/side dose was ineffective in either group (*p*’s > 0.10).

#### Forced Swim Test

**FIGURE 5:**
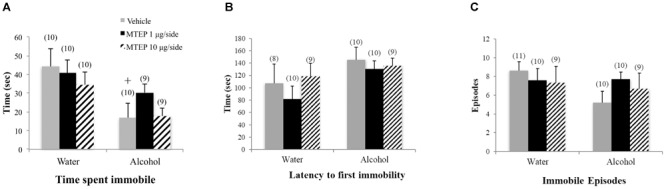
Effects of intra-AcbSh MTEP and adult-onset alcohol consumption upon behavior in the forced swim test. **(A)** Vehicle-treated alcohol drinkers specifically spent less time immobile compared to water drinkers. However, there were no treatment effects of MTEP in either water or alcohol drinkers. **(B)** There were no significant group differences in the number of immobile episodes or **(C)** latency to first immobility ^+^p < 0.05 drinking effect within same intracranial treatment group.

A significant main effect showed that alcohol-drinking animals spent less time immobile overall compared to water-drinking animals [*F*(1,52) = 9.61, *p* = 0.003; Figure 5A]. Although the interaction term was not significant, inspection of Figure 5A suggested that the effect of MTEP may depend upon the drinking history of the mice. Re-analysis of the data showed that among vehicle-treated animals, adult-onset alcohol-drinking mice spent less time immobile compared to their water-drinking controls (Tukey–Kramer: *p* = 0.008). Additionally, alcohol-drinking animals also had a longer latency to first immobility overall compared to water-drinking animals [*F*(1,50) = 4.42, *p* = 0.041; Figure 5B]. However, Tukey–Kramer comparisons did not show alcohol-related differences amongst vehicle-treated animals and neither dose of MTEP had any effect upon these two measures (*p*’s > 0.10). There were no main effects or interaction in the latency to first immobility (*p*’s > 0.10; Figure 5C).

### Intra-AcbSh MTEP Effects on the Behavior of Adolescent-Onset Drinking Mice During Protracted Alcohol Withdrawal

#### Light-Dark Box

**FIGURE 6:**
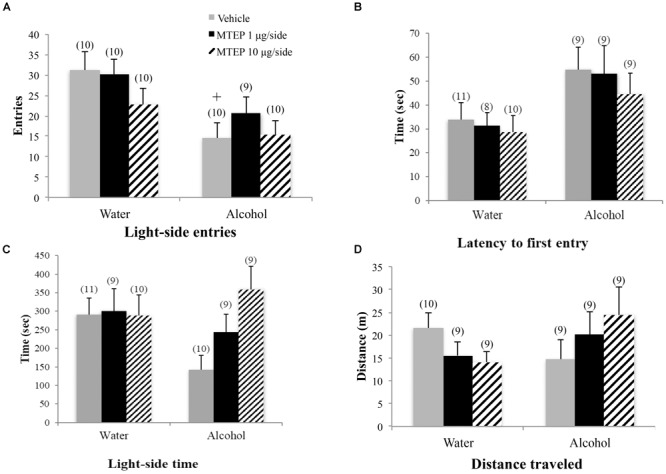
Effects of intra-AcbSh MTEP and adolescent-onset alcohol consumption upon behavior in the light-dark shuttle box test. **(A)** Vehicle-infused adolescent-onset alcohol-drinking mice made fewer light-side entries, compared to their water-drinking controls. **(B)** Alcohol-drinking mice had a significantly longer latency to first entry compared to water-drinking controls, independent of intracranial treatment. **(C)** There were no significant group differences in the total time spent on the light side or **(D)** distance traveled during this test. ^+^p < 0.05 drinking effect within same intracranial treatment group.

A significant main effect showed that adolescent-onset alcohol-drinking animals made fewer light-side entries overall compared to water-drinking animals [*F*(1,53) = 12.13, *p* = 0.001; Figure 6A]. As inspection of Figure 6A suggested that MTEP may have dose-specific effects in adolescent-onset alcohol-drinking mice. Re-analysis of these data showed that among vehicle-infused animals, alcohol-drinking mice made fewer light-side entries, compared to water-drinking controls (Tukey–Kramer’s: *p* = 0.004), but there were no effects of MTEP in either drinking group (*p*’s > 0.10). Alcohol-drinking animals also had a longer latency to first light-side entry overall compared to water-drinking animals [*F*(1,50) = 8.71, *p* = 0.005; Figure 6B]. However, re-analysis of the data using Tukey–Kramer comparisons revealed no drinking-related differences amongst vehicle-treated animals and neither dose of MTEP had an effect on latency (*p*’s > 0.10). There were no significant main effects or interactions in the total time spent on the light side or distance traveled during the test (*p*’s > 0.10; Figures 6C,D).

#### Marble-Burying

**FIGURE 7:**
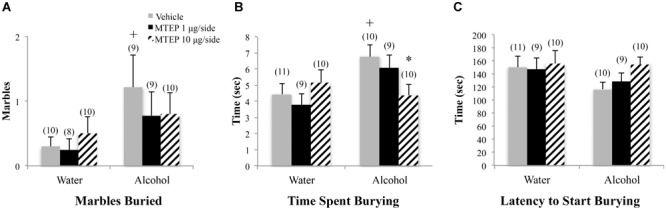
Effects of intra-AcbSh MTEP and adolescent-onset alcohol consumption upon behavior in the marble-burying test. **(A)** Among vehicle-treated animals, alcohol drinkers had a shorter latency to first immobility compared to water drinkers. **(B)** There were no significant group differences in the time spent immobile or **(C)** number of immobile episodes to begin marble burying. +p < 0.05 drinking effect within same intracranial treatment group.

A significant main effect showed that adolescent-onset alcohol-drinking animals buried more marbles overall compared to water-drinking animals [*F*(1,50) = 5.05, *p* = 0.029; Figure 7A]. Although the interaction term was not significant, inspection of Figure 7A suggested a dose-selective effect of MTEP infusion upon the behavior of alcohol-drinking adolescents. Re-analysis of the data showed that in vehicle-treated animals, alcohol-drinking mice buried more marbles, compared to water-drinking controls (Tukey–Kramer’s: *p* = 0.043). However, there were no effects of MTEP on the number of marbles buried in either drinking history group (*p*’s > 0.10). Alcohol-drinking animals also spent more time marble burying overall compared to water-drinking animals [*F*(1,53) = 4.45, *p* = 0.04; Figure 7B]. Tukey–Kramer comparisons showed that in vehicle-infused animals specifically, alcohol-drinking mice spent more time burying, compared to water-drinking controls (*p* = 0.043) and 10 μg/side MTEP significantly reduced the time spent burying by alcohol-drinking mice, compared to their vehicle-infused controls (*p* = 0.03). There were no significant group differences in the latency to start burying (*p*’s > 0.10; Figure 7C).

#### Forced Swim Test

**FIGURE 8:**
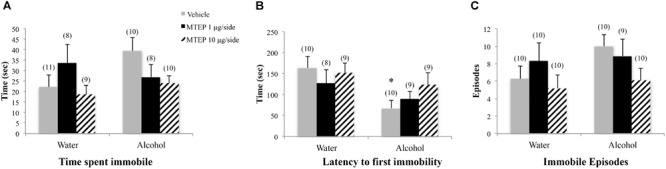
*Effects of intra-AcbSh MTEP and adolescent-onset alcohol consumption upon behavior in the forced swim test*. **(A)** No significant group differences were observed regarding the time adolescent mice spent immobile in the forced swim test. **(B)** Overall, alcohol-drinking mice exhibited a shorter latency to their first immobile episode, compared to water-drinking controls, with a significant group difference observed for the vehicle-infused mice only. MTEP did not affect this measure, irrespective of drinking history. **(C)** There were no group differences in the total number of immobile episodes. ^∗^*p* < 0.05 drinking effect within same intracranial treatment group.

There were no significant group differences in the total time spent immobile during forced swim testing as determined by ANOVA (*p*’s > 0.10; Figure 8A). A significant main effect showed that adolescent-onset alcohol-drinking animals had a shorter latency to first immobility compared to water-drinking control animals [*F*(1,49) = 7.03, *p* = 0.011; Figure 8A]. Although the interaction term was not significant, the data in Figure 8A suggested a dose-selective effect of MTEP in alcohol-drinking mice. Re-analysis of the data using Tukey–Kramer comparisons showed that in vehicle-infused animals specifically, alcohol-drinking mice had a shorter latency to first immobility compared to water-drinking controls (*p* = 0.006). However, there were no effects of MTEP treatment in either drinking group (*p*’s > 0.10). There were no significant group differences in the number of immobile episodes (*p*’s > 0.10; Figure 8C).

### Intra-AcbSh MTEP Effects on Egr1 Expression Within the AcbSh of Adult- and Adolescent-Onset Drinking Mice

**FIGURE 9:**
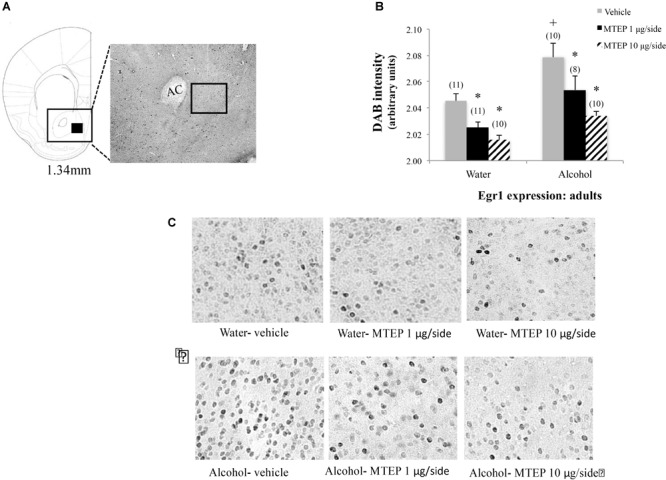
Effects of intra-AcbSh MTEP and adult-onset alcohol consumption upon Egr1 expression within the AcbSh. **(A)** An anatomical depiction of a coronal section through the striatum, highlighting the approximate size and location of the sampling region used to assay the number of Erg1+ cells within the AcbSh (black square). **(B)** Overall, adult-onset alcohol-drinking control mice exhibited higher Egr1 expression within AcbSh, relative to water-drinking controls. Both 1 μg and 10 μg/side MTEP significantly reduced Egr1 expression compared to vehicle infusion, irrespective of drinking history. **(C)** Representative micrographs of Egr1 immunostaining within the AcbSh in each of the six treatment groups. ^∗^p < 0.05 treatment effect within same drinking group, ^+^p < 0.05 drinking effect within same intracranial treatment group.

In adult-onset animals, a significant main effect showed that alcohol increased Egr1 expression within the AcbSh relative to water control animals [*F*(1,52) = 22.49, *p* < 0.001; Figures 9A–C]. There was also a significant main effect of intracranial treatment [*F*(2,52) = 14.77, *p* < 0.001]. Although no significant interaction was detected between these variables, inspection of the data in Figure 9 suggested dose-selective effects of MTEP in alcohol-experienced mice. Re-analysis of the data showed that among vehicle-treated animals specifically, adult-onset, alcohol-drinking mice exhibited higher Egr1 expression versus their water controls (Tukey–Kramer: *p* = 0.001). Compared to vehicle infusion, 1 μg/side and 10 μg/side MTEP significantly reduced Egr1 expression in both alcohol- and water-drinking animals (Tukey–Kramer comparisons, water: 1 μg/side, *p* = 0.041; 10 μg/side, *p* = 0.004. Alcohol: 1 μg/side, *p* = 0.014; 10 μg/side, *p* < 0.001).

**FIGURE 10:**
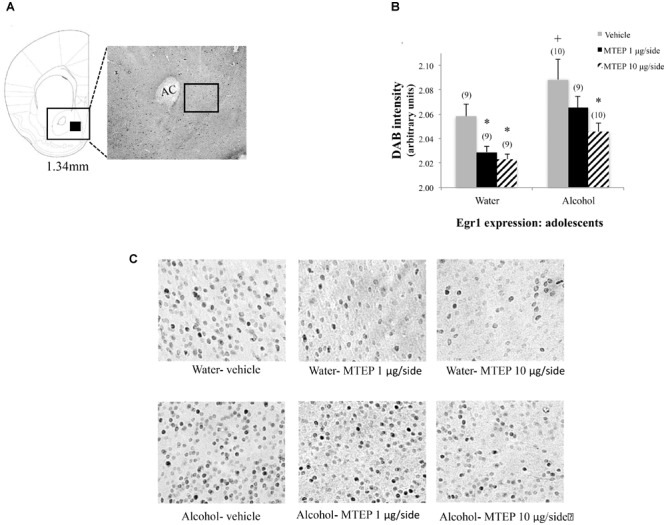
Effects of intra-AcbSh MTEP and adolescent-onset alcohol consumption upon Egr1 expression within the AcbSh. **(A)** A reference schematic of the approximate size and location of the sampling region. **(B)** Overall, vehicle-infused adolescent-onset alcohol-drinking mice exhibited higher Egr1 expression within AcbSh versus water-drinking controls. Both 1 and 10 μg/side MTEP significantly reduced Egr1 expression in water-drinking controls. However, only the reduction in Egr1 expression produced by the 10 μg/side MTEP dose was statistically significant in adolescent-onset alcohol-drinking mice. **(C)** Representative micrographs of Egr1 immunostaining within the AcbSh in each of the six treatment groups. ^∗^p < 0.05 treatment effect within same drinking group, ^+^p < 0.05 drinking effect within same intracranial treatment group.

**FIGURE 11:**
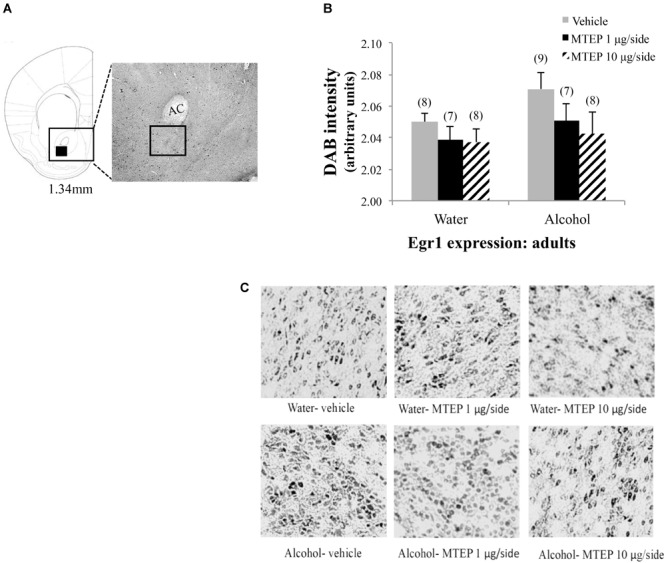
Effects of MTEP and adult alcohol experience on Egr1 expression within the AcbC. **(A)** A representative anatomical depiction of a coronal section through the striatum, highlighting (black square) the approximate size and location of the sampling region used to assay the number of Egr1+ cells within the AcbC. **(B)** There were no significant effects of adult alcohol experience or intracranial drug treatment on Egr1 expression. **(C)** Representative micrographs of Egr1 immunostaining within the AcbC in each of the six treatment groups.

**FIGURE 12:**
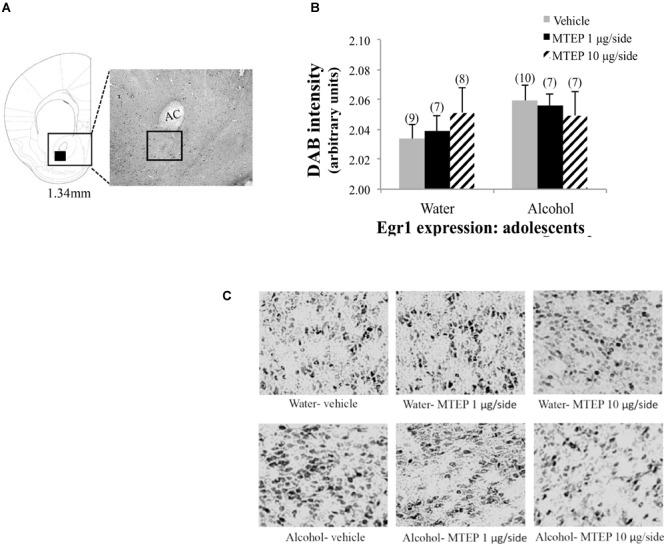
Effects of MTEP and adolescent alcohol experience on Egr1 expression within the AcbC. **(A)** A representative anatomical depiction of a coronal section through the striatum, highlighting (black square) the approximate size and location of the sampling region used to assay the number of Egr1+ cells within the AcbC. **(B)** There were no significant effects of adolescent alcohol experience or intracranial drug treatment on Egr1 expression. **(C)** Representative micrographs of Egr1 immunostaining within the AcbC in each of the six treatment groups.

In adolescent-onset animals, a significant main effect showed that alcohol increased Egr1 expression within the AcbSh relative to water control animals [*F*(1,50) = 13.42, *p* = 0.001; Figures 10A–C]. There was also a significant main effect of intracranial treatment [*F*(2,50) = 8.14, *p* = 0.001], but no interaction between these factors. As conducted for the data for adult-onset animals, Tukey–Kramer comparisons were conducted to examine the dose-selective of MTEP’s effects in adolescent-onset alcohol-drinking mice. This re-analysis showed that among vehicle-infused mice specifically, alcohol-drinking mice exhibited higher Egr1 expression, compared to their water-drinking controls (*p* = 0.009). Among water-drinking animals, both 1 μg/side and 10 μg/side MTEP reduced Egr1 expression, relative to vehicle infusion (*p* = 0.046 and *p* = 0.018, respectively). However, in adolescent-onset alcohol-drinking mice, the reduction in Egr1 expression was significant at the 10 μg/side MTEP dose (*p* = 0.003), but not at the 1 μg/side dose (*p* = 0.20). The ANOVAs revealed no significant effects of adult or adolescent alcohol drinking or intracranial drug treatment on Egr1 expression within the AcbC (*p*’s > 0.05 for main effects and interaction; Figures 11, 12).

## Discussion

Drug-induced plasticity within the AcbSh is a critical component of addiction neurobiology subserving drug reward, craving, and relapse. Although the AcbSh is a component of the emotional circuitry of the extended amygdala, relatively little is known regarding the role of the AcbSh in negative affective states during drug withdrawal. Recent correlative results suggested a causal link between the manifestation of alcohol withdrawal-induced negative affect and AcbSh expression of mGlu5 (Lee et al., 2016, 2017c). This study tested this hypothesis by determining the anxiolytic effects of inhibiting AcbSh mGlu5 using the negative allosteric modulator MTEP. Results indicate that AcbSh mGlu5 plays a modulatory role in regulating the manifestation of anxiety-like behavior during alcohol withdrawal, but that the sensitivity of this effect varies as a function of the age of alcohol-drinking onset when MTEP effects are assayed in adulthood.

### Both Adult- and Adolescent-Onset Alcohol Drinking Induces Behavioral Signs of Negative Affect

Although the alcohol intake and resulting BACs obtained on day of 11 of alcohol-access were shy of the NIAAA criterion for binge-drinking (i.e., 80 mg/dl; National Institute on Alcohol Abuse Alcoholism [NIAAA], 2004), the overall average alcohol intake exhibited by both the adult and adolescent mice in the present study was within the range reported previously by our group (Cozzoli et al., 2012, 2014a; Lee et al., 2015, 2016, 2017a,c) to result in BACs at or above the 80 mg/dl NIAAA criterion. Despite the outcome from the blood analysis, alcohol consumption was sufficient to elicit behavioral signs of negative affect, as reported previously for animals drinking at “near-binge” levels (Lee et al., 2015, 2017b). Herein, the presence of an alcohol withdrawal-induced negative affective state was confirmed based the results of omnibus ANOVAs and/or the results of planned comparisons conducted between vehicle-infused alcohol- and water-drinking mice within each age of drinking onset group. Although not every anxiety-related dependent variable was affected, adult-onset alcohol-drinking mice exhibited hyper-anxiety during early withdrawal on all 3 assays, as indicated by reduced interaction with the light side in the light-dark box, increased marble-burying activity, and decreased immobility in the forced swim test, relative to their water-drinking controls. This latter result is consistent with a series of reports from our group for adult binge- or “near-binge”-drinking mice (Lee et al., 2015, 2016, 2017a,b,c) and we have pharmacologically validated that the behavioral hyperactivity within the forced swim test manifested in early withdrawal by adult alcohol-drinking mice reflects a hyper-anxious state that can be reversed upon systemic administration of both prototypical and atypical anxiolytics, including MTEP (Lee et al., 2017b).

Also consistent with previous results from our group (Lee et al., 2016, 2017a,c, 2018) and others (Spear, 2000; Doremus et al., 2005; Spear and Varlinskaya, 2005), adolescents consumed significantly higher quantities of alcohol, compared to their adult counterparts under identical drinking conditions. As also reported recently (Lee et al., 2017c), mice with a history of adolescent-onset drinking exhibited a negative affective state during protracted withdrawal (PND 70, i.e., early adulthood), as indicated by reduced interaction with the light side in the light-dark box and increased marble burying activity in the present study. It should be noted that, while statistically higher than the behavior of water-drinking controls, the level of marble-burying behavior observed in both adult- and adolescent-onset alcohol-drinking mice was less than that reported in some of our prior work (e.g., Lee et al., 2017c). While a precise explanation for the low level of marble-burying behavior cannot be provided at this time, the levels of behavior observed herein are comparable to that observed in another one of our studies involving intracranial manipulations of the central nucleus of the amygdala (Lee et al., 2018). Not definitive by any means, the possibility exists that factors associated with surgical procedures might interfere with the expression of behavior in this assay.

As also reported previously by our group (Lee et al., 2017c) adolescent-onset alcohol-drinking mice exhibited increased immobility in the forced swim test when tested in protracted withdrawal – a phenotype opposite that observed in alcohol-drinking adults tested during early withdrawal. Increased immobility or floating in the forced swim test is conventionally interpreted as reflecting behavioral despair or a depression-like state (Porsolt et al., 1977). Consistent with the notion that adolescent-onset drinking augments depressive-like features in early adulthood, increased immobility coincides with signs of anhedonia as indicated by reduced sucrose preference (Lee et al., 2017c). Taken together, we theorize that the opposite forced swim test behavior observed in adolescent- versus adult-onset binge-drinking mice reflects age-dependent differences in alcohol withdrawal phenotype, with adult-onset mice presenting predominantly with hyper-anxiety and adolescent-onset mice presenting a combination of hyper-anxious and depressive-like features when tested in adulthood.

### Intra-AcbSh Infusion of Low Dose MTEP Reduces Withdrawal-Induced Hyper-Anxiety in Adult-Onset Mice During Early Withdrawal

The effects of intra-AcbSh MTEP exhibited an interesting drinking age-by-dose effect in our study of drug effects in adulthood. In adult-onset drinking animals, the 1 μg/side MTEP dose reduced indices of alcohol withdrawal-induced hyper-anxiety, without influencing behavior in water-drinking controls (Figures 3–5). These data extend our prior studies using systemic MTEP (Lee et al., 2017a) by indicating that the AchSh is one site involved in mediating the anxiolytic properties of this mGlu5 inhibitor during early alcohol withdrawal. Further, these neuropharmacological results provide direct cause-effect evidence that alcohol-induced increases in AchSh mGlu5 expression/function observed during early withdrawal in adult mice (Lee et al., 2016) drives components of their hyper-anxious state. As intra-AcbSh MTEP is also effective at reducing behavioral signs of heroin withdrawal-induced negative affect in adult rats (Lou et al., 2014), pharmacotherapeutics aimed at curbing excessive AcbSh mGlu5 activity may be an effective strategy for reducing the negative reinforcing properties of many drugs of abuse.

Interestingly, although an intra-AchSh infusion of higher doses of mGlu5 inhibitors (i.e., MPEP or MTEP; 10–30 μg/side) is effective at reducing binge-drinking (Cozzoli et al., 2012) and alcohol reinforcement (Besheer et al., 2009, 2010; Gass and Olive, 2009; Sinclair et al., 2012), the 10 μg/side MTEP dose did not alter anxiety-like behavior on any of our measures in adult water- or alcohol-drinking mice. This negative result for the higher MTEP dose is in line with prior reports indicating that the MTEP dose-response function may exhibit an inverted U-shaped dose-response function, with higher inhibitor doses sometimes exerting anxiogenic effects (Steckler et al., 2005; Varty et al., 2005; Belozertseva et al., 2007; Koltunowska et al., 2013; Lee et al., 2017a). The fact that both systemic treatment with, and intra-AcbSh infusion of, low MTEP doses are more effective than higher MTEP doses at reducing withdrawal-induced hyper-anxiety in adult-onset drinkers, in the absence of any overt motor side-effects or effects upon emotionality expressed by alcohol-naïve animals (Figures 3, 4; Lee et al., 2017a,b), argues the relative safety of mGlu5 antagonists. Further, the collection of data to date argue that negative allosteric modulation of mGlu5 may also have the additional benefit of minimizing the rewarding and reinforcing properties of alcohol and associated stimuli to reduce the propensity to consume more alcohol (Lominac et al., 2006; Besheer et al., 2009, 2010; Cozzoli et al., 2009, 2012; Gass and Olive, 2009; Sinclair et al., 2012).

### Intra-AcbSh Infusion of High Dose MTEP Reduces Withdrawal-Induced Hyper-Anxiety in Adolescent-Onset Mice During Protracted Withdrawal

In contrast to adult-onset drinkers, intra-AcbSh infusion of the 1 μg/side MTEP dose was ineffective at reducing any behavioral sign of the negative affective state observed during protracted withdrawal in adult mice with an adolescent-onset drinking history (Figures 6–8). Also in contrast to adult-onset drinking animals, intra-AcbSh infusion of the 10 μg/side MTEP dose exerted some anxiolytic effects (e.g., increased light-side time and decreased time spent marble-burying) in adult mice with adolescent-onset drinking experience. The age-related differences in the sensitivity of the mice to the effects of intra-AcbSh MTEP are reminiscent of our recent report, in which the MTEP dose-anxiolysis response function for adult mice with a history of adolescent-onset binge-drinking was shifted to the right of their adult-onset binge-drinking counterparts (Lee et al., 2017a). Together, these data indicate that a history of adolescent-onset alcohol-drinking reduces MTEP’s anxiolytic efficacy when assayed during protracted withdrawal. This latter finding is interesting in light of the results of an earlier report indicating that adolescent mice exhibit *greater* sensitivity to MTEP’s effects upon binge-drinking (Cozzoli et al., 2014b). In our studies, adolescent alcohol-drinking mice do not exhibit overt signs of negative affect nor do they exhibit increased Acb mGlu5 protein expression in early withdrawal (e.g., Lee et al., 2016). As such, we have focused our research efforts upon understanding the mechanisms involved in regulating the alcohol withdrawal-induced negative affective state that manifests in protracted withdrawal (i.e., in adulthood) in mice with a history of adolescent-onset drinking.

As in our prior study employing systemic MTEP administration (Lee et al., 2017a), the age of drinking-onset-related effects of intra-AcbSh MTEP are likely unrelated to the differential expression of non-specific locomotor effects as neither dose influenced the distance traveled by either alcohol- or water-drinking adolescent mice. The fact that both systemic and intra-AcbSh MTEP exert selective effects upon anxiety-like behavior also during protracted alcohol withdrawal in animals with a history of adolescent alcohol-drinking argues in favor also of its potential utility and relative safety as a therapeutic agent for minimizing emotional distress and reducing relapse probability in individuals with an early life history of problem drinking. This being said, it is important to note that sex differences are reported with respect to the ability of repeated MTEP to reduce binge-drinking, with females being more sensitive to treatment than their male counterparts (Cozzoli et al., 2014b). As our prior immunohistochemical (Lee et al., 2015), behavioral pharmacological (Lee et al., 2017a,b) and immunoblotting (Lee et al., 2016, 2017c) studies were conducted exclusively in male mice, the present study employed males as subjects only. Thus, while the shift in the MTEP dose-anxiolysis dose-response function observed in this and our earlier study (Lee et al., 2017a) argues that a history of binge-drinking during adolescence alters the developmental trajectory of mGlu5 function within the AcbSh of male mice, it remains to be determined whether or not this MTEP effect is sex-specific and/or interactions exist between sex and the age of binge-drinking onset.

While alcohol withdrawal-induced hyper-anxiety in mice with a history of binge-drinking during adulthood correlates with AcbSh mGlu5 expression (Lee et al., 2016), adolescent-onset binge-drinking does not significantly alter the total protein expression of mGlu5 within the AcbSh during either early (Lee et al., 2016) or later withdrawal (Lee et al., 2017c). Thus, the apparent shift to the right in the dose-response function for MTEP-induced anxiolysis in adult mice with a history of adolescent-onset drinking likely reflects a time-dependent increase in mGlu5 function, which could readily be attributed to increased expression of one of its major functionally relevant scaffolding proteins Homer2 (Lee et al., 2016, 2017c). While the precise mechanism(s) accounting for the age-related differences in the temporal expression of alcohol withdrawal-induced hyper-anxiety, as well as MTEP’s anxiolytic efficacy, remain to be determined, our data to date (Lee et al., 2017a, present study) have clinical ramifications for anxiolytic dosing based on the age of binge-drinking onset.

### Intra-AcbSh MTEP Does Not Affect Depressive-Like Behavior in Mice With a History of Adolescent-Onset Alcohol-Drinking

It is interesting to note that despite exerting effects on several anxiety-like measures in the light-dark shuttle box and the marble-burying tests, intra-AcbSh MTEP did not influence the behavior of male mice in the forced swim test, irrespective of the age of alcohol-drinking onset. Admittedly, the water-alcohol differences in behavior manifested on the forced swim test were smaller in this study than that reported previously by our group previously (Lee et al., 2016, 2017a,c) and this may have limited our ability to detect an MTEP effect. However, systemic MTEP treatment was also ineffective at altering swimming behavior in a recent study, in which adult-onset drinking mice exhibited robust hyper-anxiety (Lee et al., 2017a), although we have detected a significant anxiolytic effect of MTEP in a prior study of adult, alcohol-drinking, male mice (Lee et al., 2017b). While the precise reason for the assay-specific effects of intra-AcbSh MTEP remain unclear, the ineffectiveness of MTEP in the forced swim test may reflect the physical versus psychological nature of the stressor or may simply indicate that mGlu5 within the AcbSh do not regulate affective behavior in this assay. As a period of recovery is required following testing in the forced swim test, we conducted this test last in both our prior (Lee et al., 2017a) and present work (∼60 min post MTEP administration). However, the alternate explanation that MTEP’s ineffectiveness in this assay relates to the waning of drug effects/drug metabolism over time is not likely as (1) systemic and/or intracranial MTEP administration is reported to exert anti-additive effects across many different behavioral paradigms ranging in duration from 2 to 24 h (Cozzoli et al., 2012, 2014a) and (2) our Egr1 immunohistochemistry results indicate robust MTEP-induced inhibition of AcbSh activity upon completion of forced swim testing, as discussed below.

### MTEP Reduces Egr1 Indices of Neuronal Excitability in the AcbSh

When determined during adulthood, alcohol withdrawal increased Egr1 induction within the AcbSh of both adult- and adolescent-onset drinking mice, coinciding with the presence of hyper-anxiety. These results contrast with those from our earlier report in which we failed to detect withdrawal-induced changes in Egr1 expression within the AcbSh of adult mice with a 30-day history of binge-drinking (Lee et al., 2015). The discrepancies in findings might be related to a number of procedural differences between the past and present studies that include: duration of drinking history (30 vs. 14 days), age of animals when euthanized (PND 99 vs. PND 70), and drinking protocol (single 20% alcohol bottle vs. multi-bottle-choice), which render these outcomes difficult to compare. Notably, the present results are more in-line with other published studies reporting increased expression of other IEGs such as *c-fos* within the AcbSh during early withdrawal in animals with alcohol experience ranging from a single alcohol exposure (Kozell et al., 2005) to as long as 5 months of drinking (George et al., 2012). In fact, acute alcohol withdrawal has been shown to induce similar patterns of *c-fos* immunoreactivity within specific brain regions, including the AcbSh, that also respond to anxiety-provoking stimuli such as an air-puff challenge (Knapp et al., 1998) and aversive foot shocks (Duncan et al., 1996). Additionally, anxiogenic doses of caffeine and yohimbine also induce *c-fos* activation within the AcbSh (Baldwin et al., 1989; Singewald et al., 2003). Therefore, the increased Egr1 expression observed in alcohol-withdrawn mice herein is likely functionally related to the increase in anxiety-related behaviors. If so, it is particularly noteworthy that Egr1 expression was elevated in adolescent-onset drinkers even after 28 days of alcohol abstinence, providing further evidence that a 2-week history of binge-like alcohol-drinking during adolescence produces enduring changes within the activational state of the addiction-related neurocircuitry, which could drive alcohol’s incentive motivational properties and maintain compulsive alcohol-drinking (Lee et al., 2016).

Although the hyper-anxiety expressed by adult-onset alcohol-drinking mice during early withdrawal is insensitive to higher MTEP doses (Lee et al., 2017a; present study), both MTEP doses decreased Egr1 expression when infused intra-AcbSh in our adult-onset mice (Figure 9). Moreover, while MTEP did not impact emotionality or motor activity in water-drinking adult controls, MTEP dose-dependently reduced AcbSh Egr1 expression to a similar extent in adult-onset water- and alcohol-drinking animals and dose-dependently reduced AcbSh Egr1 expression in adolescent-onset water controls (Figure 10). An MTEP effect in alcohol-naïve animals was expected, as both MTEP and a related mGlu5 inhibitor, MPEP, are reported to reduce *c-fos* induction within the AcbSh (Bianchi et al., 2003; Edling et al., 2007; Besheer et al., 2009; Shin et al., 2015). However, in contrast to adult-onset alcohol-drinking animals, only the higher MTEP dose was sufficient to reduce AcbSh Egr1 expression in adolescent-onset alcohol-drinking mice. This latter result is in-line with the dose-dependency of the behavioral effects of MTEP in these animals (Figures 6–8). It is somewhat surprising that a disconnect was observed in adult-onset drinkers with respect to the effects of the 10 μg/side MTEP dose upon behavior (no effect) and AcbSh Egr1 expression (reduction). However, high doses of mGlu5 antagonist are also effective at reducing binge-alcohol drinking (Cozzoli et al., 2012), as well as the positive reinforcing properties of alcohol (Gass and Olive, 2009; Besheer et al., 2010; Sinclair et al., 2012) when infused intra-AcbSh and thus, our present immunohistochemical results may have relevance for understanding how mGlu5 inhibitors exert their “anti-alcoholism” effects.

As a final point of clarification, the focus of the present study was the AcbSh specifically, given its role in extended amygdala circuitry, and thus its potential involvement in the negative affective consequences of alcohol abuse. This being said, we also examined Egr1 expression within the adjacent AcbC and found that it was not affected by a history of alcohol drinking during either adulthood or adolescence, which is consistent with and extends our previous findings for alcohol regulation of Egr1 expression in adult animals (Lee et al., 2015). There was also no effect of intracranial drug treatment on Egr1 expression within the AcbC. This suggests either that deactivation of glutamatergic signaling within the AcbSh does not have an effect on Egr1 expression within the AcbC or that any consequent changes occur outside our sampling window of approximately 2 h post-administration. The alcohol-induced increase in cellular activation appears to be selective for the AcbSh, further supporting the potential involvement of the AcbSh in withdrawal-induced negative affect.

Many people who abuse alcohol report that anxiety reduction is a key motivator for drinking (Kushner et al., 2000) and in the clinical population, anxiety and depression during abstinence are significant sources of negative reinforcement and negative affect is one of the strongest predictors of relapse in abstinent individuals (Koob and Le Moal, 1997; Baker et al., 2004; Martinotti et al., 2008). Effectively alleviating withdrawal-induced anxiety is likely to reduce the occurrence of relapse and facilitate sobriety. Thus, additional research into the anxiolytic potential of mGlu5 antagonists, and the potential mechanisms through which these anxiolytic effects arise (e.g., direct receptor-mediated changes in excitatory or inhibitory neurotransmission and/or alternations in cannabinoid receptor 1-dependent long-term depression; including sex differences in antagonist responsiveness), could provide beneficial clinical tools for the treatment of both substance abuse and comorbid anxiety-related disorders.

## Conclusion

The results of the present study highlight an important, albeit modulatory, role for mGlu5 within the AcbSh in the age-related manifestation of hyper-anxiety during alcohol withdrawal. Adolescent-onset alcohol-drinking appeared to render mice less sensitive to MTEP’s anxiolytic effects during adulthood than adult-onset drinking, extending earlier indications that the age of drinking-onset is a critical subject factor influencing the behavioral sequelae of excessive drinking, including the severity of affective symptoms and therapeutic responsiveness. Further, we provide confirmatory evidence that heavy drinking during adolescence produces enduring behavioral and neurobiological changes within extended amygdala structures that potentially contribute to an increased vulnerability to addiction and/or affective disorders later in life.

## Author Contributions

KL and KS designed the experiments, conducted the data analyses, and composed the manuscript. KL, MiC, MaC, KS and MB performed the experiments. All co-authors edited the manuscript.

## Conflict of Interest Statement

The authors declare that the research was conducted in the absence of any commercial or financial relationships that could be construed as a potential conflict of interest.

## Footnotes

1 http://rsbweb.nih.gov/ij/
